# Toll-Like Receptor Responses to *Peste des petits ruminants* Virus in Goats and Water Buffalo

**DOI:** 10.1371/journal.pone.0111609

**Published:** 2014-11-04

**Authors:** Sakthivel Dhanasekaran, Moanaro Biswas, Ambothi R. Vignesh, R. Ramya, Gopal Dhinakar Raj, Krishnaswamy G. Tirumurugaan, Angamuthu Raja, Ranjit S. Kataria, Satya Parida, Elankumaran Subbiah

**Affiliations:** 1 Department of Animal Biotechnology, Madras Veterinary College, Tamil Nadu Veterinary and Animal Sciences University, Chennai, Tamil Nadu, India; 2 Department of Biomedical Sciences and Pathobiology, Center for Molecular Medicine and Infectious Diseases, Virginia-Maryland Regional College of Veterinary Medicine, Virginia Polytechnic Institute and State University, Blacksburg, Virginia, United States of America; 3 Animal Genetics Division, National Bureau of Animal Genetic Resources, Karnal (Haryana), India; 4 Head of FMD Vaccine Differentiation Group, The Pirbright Institute, Surrey, United Kingdom; Ella Foundation, India

## Abstract

Ovine rinderpest or goat plague is an economically important and contagious viral disease of sheep and goats, caused by the *Peste des petits ruminants* virus (PPRV). Differences in susceptibility to goat plague among different breeds and water buffalo exist. The host innate immune system discriminates between pathogen associated molecular patterns and self antigens through surveillance receptors known as Toll like receptors (TLR). We investigated the role of TLR and cytokines in differential susceptibility of goat breeds and water buffalo to PPRV. We examined the replication of PPRV in peripheral blood mononuclear cells (PBMC) of Indian domestic goats and water buffalo and demonstrated that the levels of TLR3 and TLR7 and downstream signalling molecules correlation with susceptibility vs resistance. Naturally susceptible goat breeds, Barbari and Tellichery, had dampened innate immune responses to PPRV and increased viral loads with lower basal expression levels of TLR 3/7. Upon stimulation of PBMC with synthetic TLR3 and TLR7 agonists or PPRV, the levels of proinflammatory cytokines were found to be significantly higher while immunosuppressive interleukin (IL) 10 levels were lower in PPRV resistant Kanni and Salem Black breeds and water buffalo at transcriptional level, correlating with reduced viralloads in infected PBMC. Water buffalo produced higher levels of interferon (IFN) α in comparison with goats at transcriptional and translational levels. Pre-treatment of Vero cells with human IFNα resulted in reduction of PPRV replication, confirming the role of IFNα in limiting PPRV replication. Treatment with IRS66, a TLR7 antagonist, resulted in the reduction of IFNα levels, with increased PPRV replication confirming the role of TLR7. Single nucleotide polymorphism analysis of TLR7 of these goat breeds did not show any marked nucleotide differences that might account for susceptibility vs resistance to PPRV. Analyzing other host genetic factors might provide further insights on susceptibility to PPRV and genetic polymorphisms in the host.

## Introduction

*Peste des petits ruminants* (PPR), also known as ovine rinderpest or goat plague, is an acute, highly contagious viral disease of goats and sheep, caused by the *Peste des petits ruminants* virus (PPRV), a *morbillivirus* in the family *Paramyxoviridae*. The disease is characterized by high fever, nasal and ocular discharges, pneumonia, necrotic and ulcerative lesions of the mucus membranes and inflammation of the gastro-intestinal tract [1]. PPRV infection results in great economic losses and affects productivity of sheep and goats subsequent to the global eradication of Rinderpest [2]. For example, in 2004, the economic cost of PPRV in India was estimated to be 1800 million Indian rupees (US$ 39 million) per year [2], [3]. PPRV replication and seroconversion has been demonstrated in large ruminants [4]. There is a solitary report on clinical PPRV occurring in water buffalo [5], although it has not been confirmed in later studies. In September 2004, outbreaks of PPR in Sudan affected both sheep and camels [6].

PPR is generally considered a more serious disease in goats than sheep, however, increased susceptibility of sheep, goat and outbreaks involving both sheep and goats have been equally reported [2], [3], [7], [8], [9]. Goats appear not to be affected in some outbreaks, while sheep suffer with high rates of mortality and morbidity [10]. Strain specific virulence of PPRV has been reported when the same breed of goats were experimentally infected [11], and different breeds of goat have been shown to respond differently to infection with the same virus [12]. Species-specific disease occurrence has been observed with foot and mouth disease, where cattle were highly affected while sheep had less severe infection with the virus [13]. Epizootic haemorrhagic disease virus affects cattle but sheep do not suffer from this disease [14]. It is well recognised that ducks were generally resistant to avian influenza virus (AIV) whereas chickens suffer from severe disease with rapid death following infection with highly pathogenic AIV [15]. The reason for this species specificity is unclear at present.

The natural susceptibility to PPRV in goats could be attributed to several host-derived or virus-derived factors. One such host-derived factor could be the differential presence or distribution of specific viral receptors in these species, such as the signalling lymphocyte activation molecule (SLAM) that has previously been observed to be associated with PPRV and other morbilli viruses such as measles virus and canine distemper virus [16], [17], [18]. Host immune mechanisms could also account for this differential susceptibility, although this has not been explored in detail in ruminant species or breeds.

Toll like receptors (TLR) are type 1 transmembrane proteins expressed in almost all cell types and activate the innate immune system upon sensing pathogen associated molecular patterns (PAMPs). Intracellular TLR that sense viral nucleic acids include TLR3 (double stranded RNA), TLR7 and TLR8 (single stranded RNA) and TLR9 (CpG motifs in DNA) [19]. Imiquimod and poly I:C are standard agonists used to induce TLR7 and TLR3 respectively leading to the production of inflammatory cytokines including type I interferons (IFN) and immune cell maturation [20], [21]. TLR are differentially expressed in various tissues and immune cells of water buffalo and goats, and have been shown to induce differential immune responses [22], [23]. The cell specific location and basal expression levels of TLR mRNA could indicate the natural PAMP load of that tissue as well the innate host resistance to pathogens [24].

In addition to the differential expression profiles of TLR, ligand induced downstream cytokine profiles and/or levels could also play a role in the innate disease resistance of a species or breed. For example, mastitis is an economically important inflammatory disease of the udder that has been shown to be more prevalent in the Holstein breed of cows than in Jersey cows [25]. This has been linked to temporal differences in the onset and duration of immune responses, including cytokines such as tumor necrosis factor alpha (TNFα) and IFNγ, following intramammary inoculation of pathogenic *E. coli*.

India has 23 genetically well characterized indigenous breeds of goats (http://www.icar.org.in/en/node/4688). Barbari is a common breed of goat reared for meat and milk production in northern India. Tellicherry, Kanni and Salem black are indigenous breeds of goats prevalent in southern India [26]. Outbreaks of PPR have been reported in newly introduced Barbari goats to southern India with mortality rates of 16.67% to 65.0% [27]. Recently, a severe outbreak of PPR was reported in Tellicherry breed of goats with 100% mortality in kids and 87.5% mortality in adults [28]. PPRV infection appears to be subclinical in Kanni and Salem black breeds of goats. Similarly, native water buffalo in India are resistant to PPRV. Although such anecdotal evidence and field observations on differential resistance within goat breeds and water buffalo are available, experimental evidence is lacking for the observed differences in susceptibility.

We hypothesized that the differential susceptibility of various Indian goat breeds and native water buffalo to PPRV could be related to innate immune resistance mechanisms. Infection of peripheral blood mononuclear cells (PBMC) from four breeds of goats and water buffalo resulted in differential viral replication kinetics and inflammatory cytokine profile including IFNα, IFNγ and TNFα with differential activation of TLR3 and TLR7. Analysis of single nucleotide polymorphisms (SNPs) in the complete gene sequences of TLR7 between goat breeds did not show any differences that could account for this.

## Results

### TLR3 and TLR7 mRNA expression and PPRV replication in goats

**Figure 1:**
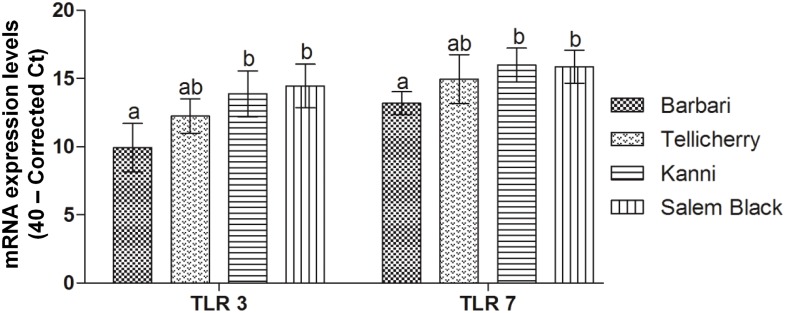
Basal expression levels of TLR3 and 7 mRNA in Barbari, Tellicherry, Kanni and Salem Black goat breeds. Significantly higher basal TLR3 (p<0.001) and TLR7 (p<0.001) mRNA expression levels observed in Kanni and Salem Black breeds, compared to Barbari. Bars with the same superscript do not differ significantly. Values represent mean ± SD of 40-corrected CT of TLR3 and 7 in nine individual animals per breed (n = 9).

**Figure 2:**
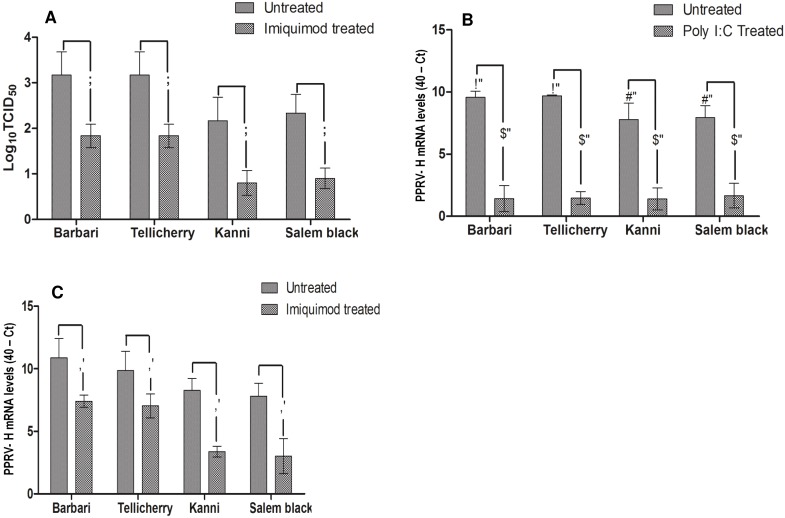
Virus replication in PBMCs stimulated with TLR3 and TLR7 agonists. a) Reduction in TCID_50_ values of PPRV in goat PBMC on imiquimod treatment. Reduction in PPRV H gene expression levels in goat PBMC on treatment with b) poly I:C and c) imiquimod. Bars with the same superscript do not differ significantly. Significance is indicated when p<0.05. Values represent mean ± SD of 40-corrected CT in 5 individual animals per goat breed. Significantly higher PPRV viral loads observed in PBMC of Barbari and Tellicherry breeds as compared to Kanni and Salem Black. Significant reduction in PPRV levels on poly I:C and imiquimod treatment in all breeds.

We examined the relative basal expression levels of TLR3 and TLR7 mRNA in naive PBMC by quantitative real-time RT-PCR (qRT-PCR) in nine individual animals per breed (n = 9). The Kanni and Salem Black breeds revealed significantly higher basal TLR3 (p<0.001) and TLR7 (p<0.001) transcripts than Barbari goats while there were no significant difference (p>0.05) between Tellicherry and Barbari breeds (Figure 1). To understand their contribution to virus replication, PBMC from each of these four goat breeds (n = 5) were infected with 1×10^3.0^ mean tissue culture infective dose (TCID_50_) of PPRV and the virus load analyzed at 24 h post infection (PI) by qRT-PCR, using primers specific to the PPRV-H gene and TCID_50_. PBMC from Barbari and Tellicherry goats supported significantly (p<0.01) higher PPRV replication than those from Kanni and Salem Black (Figure 2A) with the yields being similar in these two breeds. There was a significant reduction in virus yield by one log_10_ in Kanni/Salem Black PBMC (p<0.05) compared to Barbari/Tellicherry goats (Figure 2A).

We then examined if pre-stimulation of PBMC with the TLR agonists poly I:C and imiquimod would result in reduction in PPRV replication. A difference of about 6–8 Ct values in PPRV-H gene mRNA levels was observed between unstimulated and poly I:C treated PBMC (Figure 2B). In the case of pre-stimulation with imiquimod, this difference was about 2–3 Ct values for Barbari and Tellicherry breeds and about 4–5 Ct values for Kanni and Salem black breeds (Figure 2C). Comparison of viral load (PPRV-H gene mRNA levels and infective viral titres) in all the breeds following imiquimod treatment indicates a significant (p<0.05) reduction of viral load in Kanni and Salem black breeds than Barbari and Tellicherry breeds. TCID_50_ was not determined for poly I:C treated and PPRV infected PBMC since the 40-Ct values of PPRV H mRNA expression levels across these breeds were not significantly different (Figure 2B).

### Water buffalo PBMC are less permissive to PPRV replication

**Figure 3:**
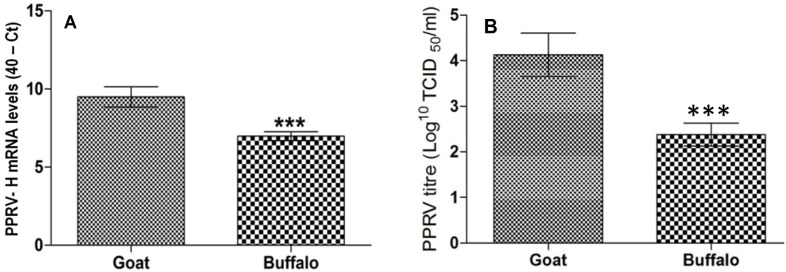
PPRV replication in goat and buffalo PBMC. Buffalo PBMC were less permissive to PPRV replication as observed by the significantly higher PPRV viral loads (P<0.001) in PBMC of goat as compared to buffalo PBMC. A) Significantly higher PPRV H gene mRNA levels and B) Significantly higher viral load in goat as compared to buffalo PBMC.

In order to investigate the species specific disease outcome, we compared the permissiveness of goat or buffalo PBMC’s to PPRV replication *in* *vitro*. Water buffalo supported significantly lower (about 4.89 fold) replication of PPRV than goat PBMC (p<0.001). Approximately 2 log_10_ difference in the virus yield was evident between buffalo and goat PBMC upon PPRV infection (Figure 3A & B).

### Cytokine mRNA expression

**Figure 4:**
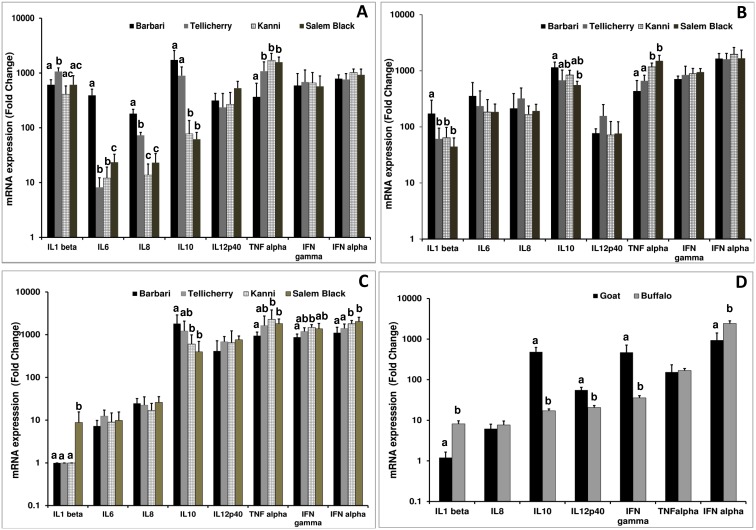
Induction of cytokine genes of goat PBMCs with TLR3 and TLR7 agonists and PPRV. A) Fold changes in mRNA expression levels of IL1β, IL6, IL8, IL10, IL12p40, TNFα, IFNγ and IFNα in goat PBMC stimulated with a) poly I:C, b) imiquimod or c) infected with PPRV d) Goat and buffalo PBMCs infected with PPRV. Fold change was determined by the 2^−ΔΔCt^ formula. An upregulation in TNFα expression levels, consistent with a downregulation in IL10 levels, is observed in Kanni and Salem Black breeds of all treatment groups. In addition, upregulation of IFNα and IFNγ was observed in the PBMC of Kanni and Salem Black breeds after PPRV infection. PPRV stimulation resulted in an upregulation of IL1β and IFNα in buffalo PBMC and IL10, IL12p40 and IFNγ in goat PBMCs. Bars with the same superscript do not differ significantly. Significance is indicated when p<0.05. Values represent mean ± SD of 5 individual animals per goat breed.

To understand the mechanistic basis for this differential permissiveness for virus replication in different goat breeds and goat vs water buffalo, we analyzed the downstream effector molecules of TLR3 and TLR7 engagement. PBMC stimulated with poly I:C, or imiquimod and further infected with PPRV were analyzed for the expression of IL1β, IL6, IL8, IL10, IL12p40, TNFα, IFNγ and IFNα mRNA (Figure 4A–D). Though upregulation of both pro- and anti-inflammatory cytokines were observed in all goat breeds, levels of TNFα was seen to be consistently higher in Kanni and Salem black breeds in all treatment groups (poly I:C, imiquimod and PPRV). This effect was prominent in the case of PPRV infected PBMC, where levels of TNFα and more importantly, IFNα and IFNγ, were significantly higher in Kanni and Salem black breeds as compared to Barbari (Figure 4C). Significant differences were not observed between these breeds and Tellicherry in the levels of pro-inflammatory cytokine mRNA. Consistent with this, levels of the immunosuppressive cytokine IL10 were significantly lower in these breeds than in Barbari (Figure 3c). Lower mRNA expression levels of IL10 in Kanni and Salem black breeds were also observed on stimulating TLR3 and TLR7 with poly I:C and imiquimod (Figure 4A, B).

**Figure 5:**
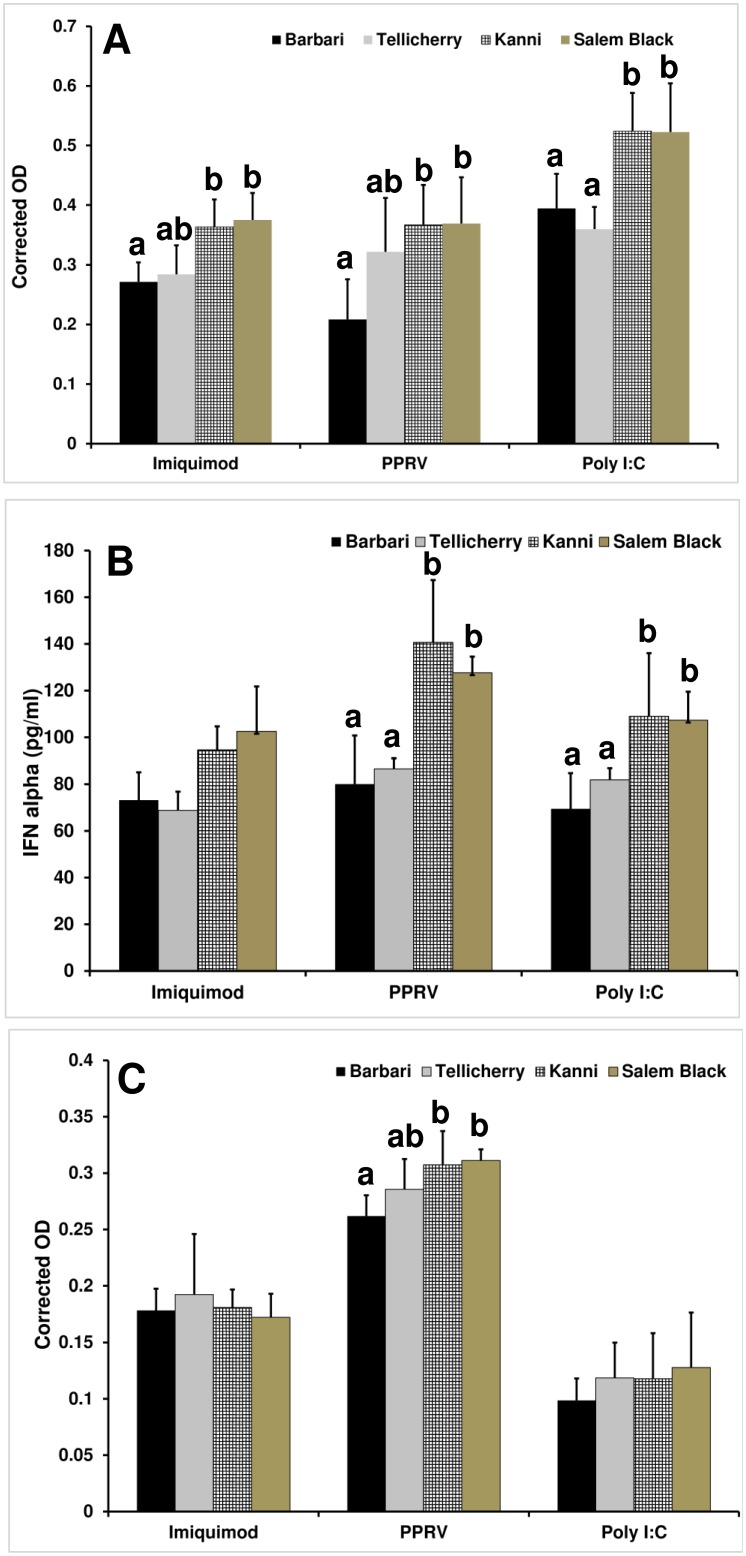
Cytokine levels in imiquimod, poly I:C or PPRV treated PBMCs. a) **TNFα,** b) IFNα, and c) IFNγ from supernatants of PPRV infected PBMC of different goat breeds. Bars with the same superscript do not differ significantly. Significance is indicated when p<0.05. PPRV infected PBMC from Kanni and Salem black breeds show higher production of TNFα, IFNα and IFNγ than Barbari. Poly I:C and imiquimod treated PBMC, similarly, show higher production of TNFα and IFNα in Kanni and Salem black breeds than in Barbari. TNFα and IFNγ levels are expressed as the corrected mean ± SD of optical density [OD] of treatment groups from which the OD of mock infected supernatants is subtracted. IFNα concentrations in the experimental samples are expressed as pg/ml.

We further validated these results by cytokine ELISA for TNFα (Figure 5A), IFNα (Figure 5B) and IFNγ (Figure 5C). Cytokine levels were similar to the mRNA expression profiles observed by qRT-PCR (Figure 5A–C). PPRV infected PBMC from Kanni and Salem black breeds had higher production of TNFα, IFNα and IFNγ than Barbari breed. Poly I:C and imiquimod treated PBMC, similarly, showed higher production of TNFα and IFNα in Kanni and Salem black breeds than in Barbari suggesting TLR3 and TLR7 engagement by PPRV.

To determine whether there are differences in the induction of pro-inflammatory cytokines between water buffalo and goats, we examined the levels of these after infecting respective PBMC with PPRV. Increased mRNA levels of IL1β and IFNα in buffalo PBMC and IL10, IL12p40 and IFNγ in goat PBMCs were observed. IFNα activation was greater in buffalo than in goats, 2360 vs 1498 fold, respectively (Figure 4D). To further confirm this observation, IFNα protein levels were assayed from culture supernatants of buffalo or goat PBMC infected with PPRV. IFNα levels in infected buffalo or goat PBMC was 86.20±4.45 and 58.13±7.48 units (p<0.01), respectively.

**Figure 6:**
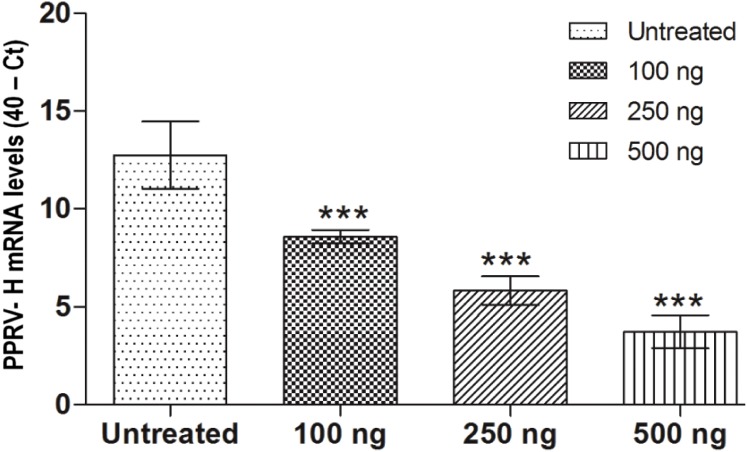
Antiviral activity of human IFNα against PPRV. Reduction in PPRV viral load was observed in Vero cells pretreated with different concentrations of human IFNα. All the doses tested significantly reduced PPRV viral load (mean ± SD of 40-Ct). Statistical significance was defined as follows: ***P<0.001.

To further define the association between differential PPRV replication and IFNα expression, the ability of IFNα in limiting the viral replication was confirmed in cells treated with human recombinant IFNα. The mean 40 - corrected Ct value of PPRV H gene expression in Vero cells without IFNα treatment was 12.10±0.42. Upon pre-treatment of Vero cells with increasing concentrations of IFNα, PPRV H gene expression decreased. Even the lowest dose of IFNα tested (100 ng/ml) could reduce the replication by 451 fold (Figure 6).

**Figure 7:**
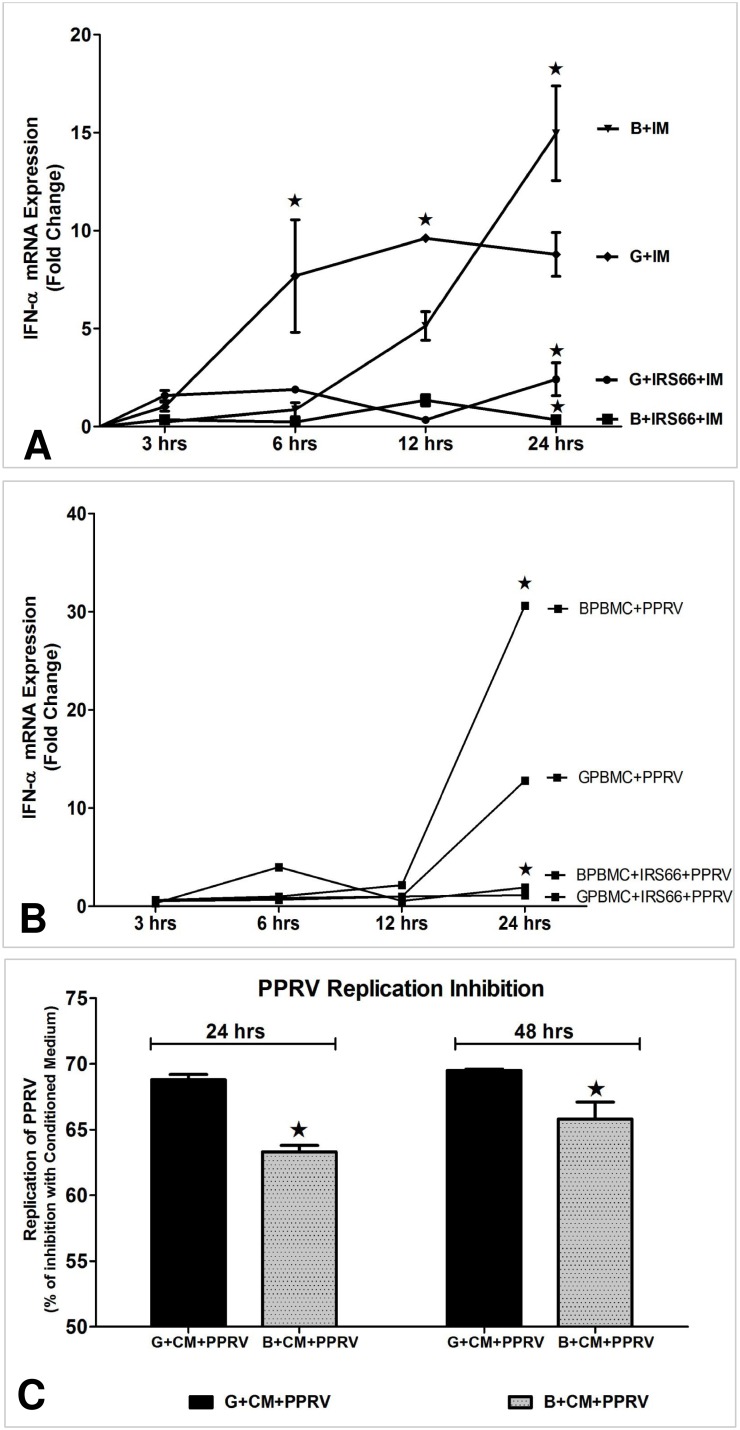
Role of type I IFN in limiting PPRV replication. a) Imiquimod or b) PPRV induced IFN-alpha mRNA expression in buffalo and goat PBMCs in the presence and absence of the TLR7 antagonist (IRS 661). Cytokine mRNA expression was quantified at 3, 6, 12 and 24 h post stimulation by qRT-PCR assays using SYBR Green chemistry. Fold change in mRNA expression induced by Imiquimod or PPRV stimulation was calculated using mock induced cytokine mRNA expression levels as a calibrator. c) PPRV replication in goat and buffalo PBMC (at 24 h and 48 h) in the presence of conditioned medium (CM) from PPRV infected cells. The expression levels of viral hemagglutinin (H) mRNA levels expressed as a percentage inhibition in viral replication in the presence of CM when compared with the control (PBMC+PPRV). Statistical significance at *P*<0.01. Values are mean ± SD of fold change/percent inhibition. B: water buffalo, G: goat, BPBMC: buffalo PBMC, GPBMC: goat PBMC.

To confirm the role of TLR7 signalling in IFNα induction by PPRV unequivocally, goat and buffalo PBMCs were treated with a predetermined optimum concentration (10 µg/ml) of a TLR7 antagonist IRS 661 [] 24 h prior to imiquimod treatment or PPRV infection. IFNα mRNA levels were significantly reduced by IRS 661 treatment, when PBMC were stimulated either with Imiquimod or PPRV (Figure 7A, B). To further confirm the role of TLR7 induced IFNα in limiting virus replication, conditioned medium (CM) from PPRV infected cells were used to pre-treat PBMC before virus infection (Figure 7C). The percent inhibition in virus replication was significantly higher (p<0.01) in CM pre-treated buffalo PBMC than goat PBMC (Figure 7C).

### Genetic polymorphisms in TLR7 across goat breeds

To determine whether the differential IFN and pro-inflammatory cytokine production between Kanni/Salem vs Barbari/Tellicherry breeds of goats are dependent on single nucleotide polymorphisms (SNPs) in TLR genes, we examined the complete gene sequence of TLR7. The TLR7 gene was amplified and seven overlapping PCR amplicons were sequenced to obtain the full length TLR7 gene using the goat TLR7 sequence from GenBank as a template (Accession # GU289401). The sequences obtained for Barbari, Tellicherry and Kanni goat breeds were submitted to GenBank (Accession # KC127658, KC127659, KC127660, KC127661, KC127662, KC127663, KC127657). Nucleotide variations were detected across goat breeds. Sequence analysis revealed five nucleotide changes in the TLR7 coding region and two nucleotide changes in the 3′ untranslated region (3′UTR). Changes were observed at nucleotide positions 777 (C/T in Salem while C/C in other breeds), 2082 (A/A in Salem while A/G in other breeds), 2730 (C/T in Barbari while C/C in other breeds), 3006 (A/A in Tellicherry while A/G in other breeds) and 3069 (G/A in Barbari while G/G in other breeds) that corresponded to amino acid positions 259, 694, 910, 1002 and 1023. However, all of these changes were synonymous and no amino acid changes were observed between different goat breeds. In addition, two changes in the 3′UTR, at nucleotide positions 151 (G/A in Barbari and Salem while G/G in other two breeds) and 161 (G/A in Barbari while G/G in other breeds) were also observed.

## Discussion

Following eradication of rinderpest, focus has shifted to PPRV as a potential virus for eradication efforts. The single serotype of PPRV, availability of an efficacious live attenuated vaccine, sensitive diagnostic tests and the economic impact of this disease, coupled with its ability to spread into new geographical regions, render it an attractive target [30]. PPR was first reported in India in sheep with a lineage IV virus in 1989 [31], with no further reports until 1996, when a massive outbreak occurred in goats throughout northern India with a lineage IV virus [32]. Three live attenuated vaccines, specific to lineage IV PPRV strains have so far been tested in India [33].

Goats have been reported to be more susceptible to PPRV than sheep, while cattle and buffalo do not contract clinical disease [5], [32], [34]. Increased mortality in lambs/kids, and increased susceptibility of West African goats, especially dwarf goats, compared to their European counterparts have been documented [11]. Differential susceptibility of goat breeds within India have also been reported [35]. Host genetic factors, in particular the major histocompatibility complex (MHC) genes, may influence susceptibility to disease. Virus recognition can be influenced by genetic mutations in the interaction domains between virus and host receptors. In particular, non-MHC genetic variations in host TLR may cause reduced pathogen recognition and hamper innate immune activation [36]. Studies on Maedi-Visna infection in sheep indicate that breed dependent susceptibility to the disease as well as individual susceptibility within the breed may be defined by specific polymorphisms in TLR7 and TLR8 genes [37]. In a related morbillivirus, SNPs in TLR 3, 4, 5, 6 and associated signalling molecules like Myeloid differentiation primary response gene 88 (MyD88) and MD2 affected immune responses to the measles vaccine in human subjects [38].

Single and double stranded RNA are recognized by TLR7/8 and TLR3, respectively. TLR3 is a key sensor of viral infection, as most viruses will produce dsRNA at some stage of its life cycle. TLR7 is highly expressed in immune cells like plasmacytoid dendritic cells (pDC), which produce substantial amounts of type I IFNs in response to viral RNA. In our study, the basal levels of TLR3 and TLR7 were significantly higher in the PBMCs of PPRV resistant goat breeds, Kanni and Salem Black. Engagement of both TLR3 and TLR7 with the synthetic ligands poly I:C and imiquimod respectively, led to the suppression of PPRV RNA and infectious virus yield in PBMC of goats. This indicates that TLR3 and 7 play a role in the recognition of PPRV RNA by goat PBMC, though the role of cytosolic RNA sensors like Retinoic acid-inducible gene 1 (RIGI) and Melanoma differentiation-associated protein 5 (MDA5) have not been analyzed in this study and cannot be ruled out. Almost complete abrogation of viral gene expression was observed after stimulation by poly I:C. This may be because poly I:C can also be recognized by other sensors, including RIGI, MDA5 and Protein kinase R (PKR) [39].

If factors other than the receptor expression for PPRV determine clinical disease, this should be at the level of virus replication and clearance by innate and adaptive responses. The cell surface receptor for PPRV, the SLAM is expressed at lower levels in buffalo than goats [16]. However, we did see virus replication in infected PBMC although at considerably reduced levels suggesting that the cells of water buffalo are permissive to PPRV infection. Therefore, we questioned whether these differences are reflected at the level of multi-cycle virus replication. PPRV replication in water buffalo PBMC was significantly lower than in goats, possibly because of enhanced type I IFN production in these species upon virus infection. PPRV replication in human IFNα pre-treated Vero cells or in PBMC pre-treated with CM from virus infected cells was significantly lower even at very low doses, confirming the role of type I IFN in limiting virus replication. Human IFNα has been shown to effectively suppress the replication of bovine viral diarrhea virus and bovine parainfluenza virus [40], [41].

Cytokines play a pivotal role in the induction and modulation of immunological responses. TLR signaling events lead to the activation of nuclear factor kapp-light-chain-enhancer of activated B cells (NFκB) and interferon regulatory factor (IRF), which switch on expression of a specific panel of pro-inflammatory cytokines and chemokines such as TNFα, IL6, IL8 and Regulated on activation, Normal T cells (RANTES) [19]. Activation of TLR by viruses also results in the production and release of type I IFNs [39]. TLR3 and TLR7 engagement by synthetic ligands lead to cytokine expression profiles similar to PPRV infection except for a weak IL1β, IL6 and IL8 production in goat PBMC. A predominantly inflammatory cytokine repertoire, with expression of TNFα, IFNα and IFNγ was observed at both mRNA and protein levels. Thus, it could be inferred that TLR engagement upon PPRV infection results in inflammatory cytokine production via the canonical NFκB pathway and type I IFN production via the activation of IRFs [42]. Stimulation of TLR7 with synthetic RNA oligonucleotides has earlier been shown to induce production of IL-12, TNFα and IFNγ in PBMC of cattle [43]. Interestingly, in our study, IFNγ levels were higher in PPRV infected PBMC, compared to the engagement of TLR3/7 by their respective agonists. IFNγ production by NK cells can be induced by IL12 secreted by TLR stimulated DCs [44]. In buffalos, approximately 1.5 fold higher levels of IFNα at mRNA and protein levels were induced in PBMC compared to goats after infection with PPRV suggesting that type I IFN may play a role in limiting virus replication in buffalo. Further, we found that TLR7 mediated IFNα production is critical because TLR7 antagonist inhibited IFNα production both in Imiquimod or PPRV-treated goat and buffalo PBMC. This effect was more prominent in buffalo PBMC suggesting that TLR7 mediated IFNα production determines PPRV replication efficiency in this species.

Consistent with the inflammatory cytokine environment induced by PPRV infection, expression of the immunomodulatory cytokine IL10 was also observed, but its levels were high in the PPRV susceptible goat breeds, Barbari and Tellicherry. IL10 is a key regulatory cytokine with immunosuppressive properties that helps to regulate an uncontrolled inflammatory response [45]. In addition to preventing the maturation of antigen presenting cells, IL10 can also regulate the proliferation and differentiation of Th1 cells, which induces T cell-dependent suppression of antiviral responses [46], [47]. Dexamethasone, a well-known immunosuppressive drug, induces immunosuppression by altering the expression levels of IL10 and TNFα [48]. Experimental immunosuppression of goat with dexamethasone and challenge with virulent PPRV indicated that, immunosuppressed goats had a shorter viremia, more extensive and severe disease advancement, significant decrease in the number of leucocytes, high antigen load in various organs and higher mortality rate than the non-immunosuppressed goats [49], [50]. Taken together, it appears that a higher basal expression of TLR3 and TLR7 in Kanni and Salem breeds may correlate with increased inflammatory cytokine expression with lower levels of immunomodulatory cytokines leading to an enhanced antiviral state thus affording reduced susceptibility to PPRV infection. Similarly, in buffalo, the TLR-7 mediated type I IFN response upon infection may afford resistance to PPRV.

The goat TLR7 gene is 3.4 Kb long, with a 3141 nucleotide open reading frame (ORF), coding for 1046 amino acids. Nucleotide sequence homology studies have shown a close relationship with other ruminant species, particularly sheep TLR7 [51]. Earlier studies in 12 Indian goat breeds have shown a total of 22 polymorphic sites, out of which 19 were present within the coding region and three in the 3′UTR [41]. The Toll/interleukin-1 receptor (TIR) domain sequence is highly conserved between species, as it plays a crucial role in TLR downstream signaling [52]. In our study, sequence analysis revealed five nucleotide changes in the TLR7 coding region and two nucleotide changes in the 3′UTR. All the changes were synonymous and it is difficult to establish a correlation with specific SNP and altered susceptibility to PPRV in the goat breeds examined. There were no differences in the leucine repeat regions of TLR7 between different breeds of goats. Though, we were unable to demonstrate a positive association between SNP and differential susceptibility to PPRV in the goat TLR7 gene, analyses of other immune genes including TLR3 and TLR4 may indicate the relationship between susceptibility to PPRV infection and genetic polymorphisms in the host. Earlier studies in buffalo TLR7 gene have also reported four different polymorphic positions (A/G-1400, A/G-1480 (D234N), C/T-2029 (L417F), A/G-2640), of which two were non synonymous SNP’s in leucine rich repeats (LRR) [53]. Given the presence of SNP’s in LRR of buffalo TLR7 gene, which is responsible for sensing the PAMP’s, these SNP’s could be associated with differential sensing of PPRV. Further studies are required to correlate the reported SNP’s in buffalo TLR7 with observed differential response of buffalo PBMC to PPRV.

We also found a role for basal and induced TLR3 expression levels in PPRV infection in our study. In addition to TLR, variations in downstream intracellular signaling molecules such as MyD88 and MD2 may also play a role. In conclusion, our studies suggest that higher basal levels of TLR3/7 and augmented innate antiviral immune responses upon infection may afford resistance to PPRV infection in Kanni and Salem breeds of goats compared to Barbari and Tellicherry breeds. Compared to goats, elevated type I IFN levels after PPRV infection in water buffaloes may afford reduced virus replication and possibly early virus clearance. LRR prediction revealed goat TLR7 has 21 LRRs and buffalo TLR7 has 15 LRRs. The difference in LRR numbers could be a critical factor in determining the signaling responses of goat and buffalo TLR7. Future studies may provide insights into understanding the immunogenetic mechanisms underlying variations in the immune response to PPRV.

## Materials and Methods

### Animals, virus and reagents

All animal studies have been conducted as approved by the Ethics Committee of the Tamil Nadu Veterinary and Animal Sciences University, Chennai-600 051, India. Apparently healthy, 12–18 month old, Barbari, Tellicherry, Kanni and Salem Black breeds of goats of either sex were maintained under similar conditions at the Livestock Research Station, Tamil Nadu Veterinary and Animal Sciences University, India. These animals were not vaccinated against PPRV and had no record of any other disease during the course of the study. PBMC from nine animals of each breed were used for TLR mRNA expression analysis, while PPRV infection and TLR stimulationstudies were carried out on PBMC from five animals of each breed. Live attenuated PPRV (strain AR-87) was obtained from the department of veterinary microbiology, Madras Veterinary College, India and has been described elsewhere [31]. Imiquimod R837 (2.5 µg/ml) and polyionosinic-polycytidylic acid, a synthetic analog of dsRNA (poly I:C) (25 µg/ml) (InVivoGen, San Diego, CA) were diluted in endotoxin-free water. Aliquots were tested by the E-toxate kit (*Limulus*am ebocyte lysate assay, Sigma Aldrich, St. Louis, MO) and found to be free of endotoxins.

### TLR stimulation and PPRV infection

Blood was collected aseptically from the jugular vein into sterile ethylene diamine tetra acetic acid (EDTA) coated vacutainer tubes (Becton Dickenson, Cambridge, UK) and processed for PBMC isolation. Briefly, 5 ml of anti-coagulated blood was diluted in equal volumes of RPMI-1640 (Invitrogen, Paisley, UK) medium containing antibiotic and antimycotic solution, overlaid on 2.5 ml of Histopaque, specific gravity 1.077 (Sigma Aldrich, St. Louis, MO) and centrifuged at 1500×g for 25 min. Mononuclear cells were collected from the interface and washed three times in RPMI-1640 by centrifugation at 200×g for 10 min. Viability was determined by trypan blue dye exclusion method. PBMC were stimulated with predetermined doses of TLR3 and TLR7 agonists, poly I:C and imiquimod (R837), respectively. Cells were harvested at 24 h for cytokine transcript analysis.

In a separate experiment, TLR ligand stimulated PBMC were infected with 10^3.0^ TCID_50_ of PPRV. Virus yields from TLR 3/7 stimulated and un-stimulated PBMC were assessed at 24 h PI by SyBr Green quantitative real time reverse transcription polymerase chain reaction (qRT-PCR), using primers specific for the PPRV-H gene [35]. Infective virus in the supernatants of PPRV infected PBMC cultures were determined on Vero cells and expressed as TCID_50_/mL [54].

### Analysis of basal TLR transcript levels

Total RNA from PBMCs was extracted using TRI reagent solution (Sigma-Aldrich, St. Louis, MO) as permanufacturer’s instructions. RNA concentration and purity was determined using the BioPhotometer plus (Eppendorf, Hamburg, Germany). Two µg of total RNA was reverse transcribed with Oligo (dT)_18_ primers using the High Capacity cDNA Archive kit (Applied Biosystems, Carlsbad, CA). Basal expression levels of TLR3 and TLR7 mRNA in PBMC were determined using gene-specific primers and TaqMan probes (FAM-NFQ) (Applied Biosystems, Carlsbad, CA) (Table 1). qRT-PCR was performed in triplicate under the following cycle conditions, 2 min at 50°C, 10 min at 95°C and 40 cycles of 95°C for 15 sec and 60°C for 1 min (Applied Biosystems 7500 Real time PCR System, Carlsbad, CA).

**Table 1:** TaqMan primer/Probe sequences for assessing basal expression levels of TLR3 and TLR7 mRNA.

Target gene	Primer/Probe sequence 5′-3′ (Forward/Reverse)	Efficiency	Slope	Accession number
TLR7	GCTCCAAATGCCCATGTGATT	0.953	−3.43	HQ263216
	AGGAATACCTCCAGGAATTTCTGTCA			
	FAM 5′CTGCACAGACAAACTT 3′ NFQ			
TLR3	GTCCTTGACCTCGGCCTTAA	0.976	−3.38	H263210
	CCCCATTCTTGGCCTGTGA			
	FAM 5′ TTCTTGCCCAATTTCA 3′ NFQ			
GAPDH	GGCGCCAAGAGGGTCAT	0.942	−3.46	AJ431207
	GTTCACGCCCATCACAAACAT			
	FAM 5′CTTCTGCTGATGCCCC3′ NFQ			

### Cytokine transcripts after stimulation with TLR agonists or infection with PPRV

Cytokine gene expression levels were compared by SyBr Green qRT-PCR using gene specific primers (Applied Biosystems, Carlsbad, CA) (primer sequences available upon request). Un-stimulated PBMC were used as control. Corrected Ct was calculated as:

Ct + (Nt – Ct’)×S/S’, where Ct is the mean sample Ct, Nt is the mean of the house keeping genes GAPDH/actin from the control group, Ct′ is the mean of the GAPDH/actin from treatment, S is the target gene slope, and S’ is the GAPDH/actin slope. The slope values were calculated using serial dilutions of cDNA and the respective Ct values for each dilution and PCR efficiency (E = 10^−1^/slope) was determined. Results were expressed as 40-Ct values [54], [55]. Changes in cytokine expression were expressed as fold change (2^−ΔΔCt^) over the respective basal levels of mock-induced PBMCs after normalizing for the endogenous control gene and using the corrected Ct.

### Differential Enzyme linked immunosorbent assay (ELISA) for TNFα, IFNα and IFNγ

Antigen capture ELISA kits for TNFα (AbDSerotec, Kidlington, UK), IFNα (Mabtech, Sweden) and IFNγ (CUSABIO Biotech, China) were used to determine cytokine concentrations in the culture supernatants of TLR ligand stimulated and/or PPRV infected PBMC. ELISA was performed according to the manufacturer’s instructions and values were obtained spectrophotometrically on an ELISA reader (Epoch Micro-Volume Spectrophotometer System, Biotek, Winooski, VT) at 492 nm. Mock infected cell culture supernatant served as a control. TNFα and IFNγ levels were expressed as the corrected optical density [OD] of TLR-ligand stimulated or PPRV infected culture supernatants from which the OD of mock infected supernatants is subtracted. IFNα concentrations in the experimental samples were extrapolated from the values generated from standards.

### Detection of single nucleotide polymorphisms in TLR7 gene

Blood samples were collected from Barbari, Tellicherry, Kanni and Salem black breeds of goat and genomic DNA was isolated using the Blood DNA isolation kit (Biobasic, USA). The concentration of extracted DNA was determined using biophotometer plus (Eppendorf, Germany). Seven pairs of overlapping primers were designed to amplify the full-length TLR7 gene (primers available upon request) and the PCR fragments were directly sequenced in both directions using the Big Dye Terminator v3.1 cycle sequencing kit (Applied Biosystems, Carlsbad, CA). Sequences were assembled into a 3.4 kb contig, which contained a 3141 bp open reading frame. Sequence contigs of TLR7 from each animal were further subjected to multiple alignments to identify nucleotide variations, using the Lasergene software (DNASTAR, Madison, WI). Heterozygous nucleotides were scored manually across samples from different breeds by visualizing the individual chromatogram in Chromas Lite 2.01 (Technelysium, Queensland, Australia). Each polymorphic nucleotide was further analyzedfor its amino acid position and change.

### Statistical analysis

Statistical analysis was performed with the GraphPad Prism software. The 40-corrected Ct values of TLR3 and TLR7 mRNA, fold changes in the ligand induced cytokine mRNA expression, PPRV H gene levels and virus yield estimated by TCID_50_ determination was compared by two-way ANOVA with Bonferroni test for multiple comparisons. ELISA values for each cytokine and PPRV-H gene levels upon IFNα treatment were compared by one-way ANOVA with Bonferroni test for multiple comparisons. Means were considered significantly different when the p value was <0.05.

## References

[1] Lefevre PC, Diallo A (1990) Peste des petits ruminants. Rev Sci Tech 9: 935–981. 2132714 10.20506/rst.9.4.532

[2] Singh RP, Saravanan P, Sreenivasa BP, Singh RK, Bandyopadhyay SK (2004) Prevalence and distribution of peste des petits ruminants virus infection in small ruminants in India. Rev Sci Tech 23: 807–819. 15861876 10.20506/rst.23.3.1522

[3] Banyard AC, Parida S, Batten C, Oura C, Kwiatek O, et al (2010) Global distribution of peste des petits ruminants virus and prospects for improved diagnosis and control. J Gen Virol 91: 2885–2897. 20844089 10.1099/vir.0.025841-0

[4] Khan HA, Siddique M, Sajjad ur R, Abubakar M, Ashraf M (2008) The detection of antibody against peste des petits ruminants virus in sheep, goats, cattle and buffaloes. Trop Anim Health Prod 40: 521–527. 18716909 10.1007/s11250-008-9129-2

[5] Govindarajan R, Koteeswaran A, Venugopalan AT, Shyam G, Shaouna S, et al (1997) Isolation of pestes des petits ruminants virus from an outbreak in Indian buffalo (Bubalus bubalis). Vet Rec 141: 573–574. 9423239 10.1136/vr.141.22.573

[6] Saeed IK, Ali YH, Khalafalla AI, Rahman-Mahasin EA (2010) Current situation of Peste des petits ruminants (PPR) in the Sudan. Trop anim Health Prod 42: 89–93. 19548103 10.1007/s11250-009-9389-5

[7] Roeder PL, Abraham G, Kenfe G, Barrett T (1994) Peste des petits ruminants in Ethiopian goats. Trop Anim Health Prod 26: 69–73. 7941031 10.1007/BF02239901

[8] Taylor WP, Abegunde A (1979) The isolation of peste des petits ruminants virus from Nigerian sheep and goats. Res Vet Sci 26: 94–96. 472495

[9] Taylor WP, Diallo A, Gopalakrishna S, SreeramaluP, Wilsmore AJ, et al (2002) Peste des petits ruminants has been widely present in southern India since, if not before, the late 1980s. Prev Vet Med 52: 305–312. 11849724 10.1016/s0167-5877(01)00254-9

[10] Yesilbag K, Yilmaz Z, Golcu E, Ozkul A (2005) Peste des petits ruminants outbreak in western Turkey. Vet Rec 157: 260–261. 16127137 10.1136/vr.157.9.260

[11] Couacy-Hymann E, Bodjo C, Danho T, Libeau G, Diallo A (2007) Evaluation of the virulence of some strains of peste-des-petits-ruminants virus (PPRV) in experimentally infected West African dwarf goats. Vet J 173: 178–183. 16310383 10.1016/j.tvjl.2005.08.020

[12] Diop M, Sarr J, Libeau G (2005) Evaluation of novel diagnostic tools for peste des petits ruminants virus in naturally infected goat herds. Epidemiol Infect 133: 711–717. 16050518 10.1017/s0950268805003729 PMC2870300

[13] Anderson EC, Anderson J, Doughty WJ, Drevmo S (1975) The pathogenicity of bovine strains of foot-and-mouth disease virus for impala and wildebeest. J Wildl Dis 11: 248–255. 167208 10.7589/0090-3558-11.2.248

[14] Kedmi M, Levi S, Galon N, Bomborov V, Yadin H, et al (2011) No evidence for involvement of sheep in the epidemiology of cattle virulent epizootic hemorrhagic disease virus. Vet Microbiol 148: 408–12. 20956061 10.1016/j.vetmic.2010.09.015

[15] Kuchipudi SV, Dunham SP, Nelli R, White GA, Coward VJ, et al (2012) Rapid death of duck cells infected with influenza: a potential mechanism for host resistance to H5N1. Immunol Cell Biol 90: 116–123. 21423263 10.1038/icb.2011.17 PMC3257048

[16] Pawar RM, Dhinakar Raj G, Balachandran C (2008a) Relationship between the level of signaling lymphocyte activation molecule mRNA and replication of Peste-des-petits-ruminants virus in peripheral blood mononuclear cells of host animals. Acta virol 52: 231–236. 19143479

[17] Sidorenko SP, Clark EA (2003) The dual-function CD150 receptor subfamily: the viral attraction. Nature Immunol 4: 19–24. 12496974 10.1038/ni0103-19

[18] Tatsuo H, Ono N, Yanagi Y (2001) Morbilliviruses use signaling lymphocyte activation molecules (CD150) as cellular receptors. J Virol 75: 5842–5850. 11390585 10.1128/JVI.75.13.5842-5850.2001 PMC114299

[19] Kawai T, Akira S (2007) TLR signaling. Sem Immunol 19: 24–32. 10.1016/j.smim.2006.12.004 17275323

[20] Booth JS, Buza JJ, Potter A, Babiuk LA, Mutwiri GK (2010) Co-stimulation with TLR7/8 and TLR9 agonists induce down-regulation of innate immune responses in sheep blood mononuclear and B cells. Dev Comp Immunol 34: 572–578. 20051250 10.1016/j.dci.2009.12.018

[21] Hemmi H, Kaisho T, Takeuchi O, Sato S, Sanjo H, et al (2002) Small anti-viral compounds activate immune cells via the TLR7 MyD88-dependent signaling pathway. Nature Immunol 3: 196–200. 11812998 10.1038/ni758

[22] Vignesh AR, Dhanasekaran S, Raj GD, Balachandran C, Pazhanivel N, et al (2012) Transcript profiling of pattern recognition receptors in a semi domesticated breed of buffalo, Toda, of India. Vet Immunol Immunopathol 147: 51–9. 22521945 10.1016/j.vetimm.2012.02.009

[23] Tirumurugaan KG, Dhanasekaran S, Raj GD, Raja A, Kumanan K, et al (2010) Differential expression of toll-like receptor mRNA in selected tissues of goat (*Capra hircus*). Vet Immunol Immunopathol 133: 296–301. 19748133 10.1016/j.vetimm.2009.08.015

[24] Menzies M, Ingham A (2006) Identification and expression of Toll-like receptors 1–10 in selected bovine and ovine tissues. Vet Immunol Immunopathol 109: 23–30. 16095720 10.1016/j.vetimm.2005.06.014

[25] Bannerman DD, Kauf AC, Paape MJ, Springer HR, Goff JP (2008) Comparison of Holstein and Jersey innate immune responses to Escherichia coli intramammary infection. J Dairy Sci 91: 2225–2235. 18487645 10.3168/jds.2008-1013

[26] Joshi MB, Rout PK, Mandal AK, Tyler-Smith C, Singh L, et al (2004) Phylogeography and origin of Indian domestic goats. Mol Biol Evol 21: 454–462. 14660684 10.1093/molbev/msh038

[27] Rita NP, Gopu S, Baegan S (2008) Barathidasan (2008) Clinical management in an outbreak of peste des petits ruminants in Barbari goats. Vet World 1: 81–82.

[28] Roy P, Vairamuthu S, Thangavelu A, Chitradevi S, Purushothaman V, et al (2010) An outbreak of peste des petits ruminants among Thelichery breed of goats. Int J Appl Res Vet Med 8: 155–160.

[29] Barrat FJ, Meeker T, Chan JH, Guiducci C, Coffman RL (2007) Treatment of lupus-prone mice with a dual inhibitor of TLR7 and TLR9 leads to reduction of autoantibody production and amelioration of disease symptoms. Eur J Immunol 37: 3582–6. 18034431 10.1002/eji.200737815

[30] Baron MD, Parida S, Oura CA (2011) Peste des petits ruminants: a suitable candidate for eradication? Vet Rec 169: 16–21. 21724765 10.1136/vr.d3947

[31] Shaila MS, Purushothaman V, Bhavasar D, Venugopal K, Venkatesan RA (1989) Peste des petits ruminants of sheep in India. Vet Rec 125: 602. 2609485

[32] Nanda YP, Chatterjee A, Purohit AK, Diallo A, Innui K, et al (1996) The isolation of peste des petits ruminants virus from northern India. Vet Microbiol 51: 207–216. 8870184 10.1016/0378-1135(96)00025-9

[33] Saravanan P, Sen A, Balamurugan V, Rajak KK, Bhanuprakash V, et al (2010) Comparative efficacy of peste des petits ruminants (PPR) vaccines. Biologicals 38: 479–485. 20199873 10.1016/j.biologicals.2010.02.003

[34] Mornet P, Orue J, Gilbert Y, Thiery G, Mamadou S (1956) La peste des petite ruminants en Afrique occidentale française ses rapports avec la peste bovine. Revue d’élevage et de médecine vétérinaire des pays tropicaux 9: 313–342.

[35] Pawar RM, Raj GD, Kumar TM, Raja A, Balachandran C (2008b) Effect of siRNA mediated suppression of signaling lymphocyte activation molecule on replication of peste des petits ruminants virus in vitro. Virus Res 136: 118–123. 18550191 10.1016/j.virusres.2008.04.026 PMC7127705

[36] Schroder NW, Schumann RR (2005) Single nucleotide polymorphisms of Toll-like receptors and susceptibility to infectious disease. Lancet Infect Dis 5: 156–164. 15766650 10.1016/S1473-3099(05)01308-3

[37] Mikula I, Bhide M, Pastorekova S, Mikula I (2010) Characterization of ovine TLR7 and TLR8 protein coding regions, detection of mutations and Maedi Visna virus infection. Vet Immunol Immunopathol 138: 51–59. 20638136 10.1016/j.vetimm.2010.06.015

[38] Dhiman N, Ovsyannikova IG, Vierkant RA, Ryan JE, Pankratz VS, et al (2008) Associations between SNPs in toll-like receptors and related intracellular signaling molecules and immune responses to measles vaccine: preliminary results. Vaccine 26: 1731–1736. 18325643 10.1016/j.vaccine.2008.01.017 PMC2292110

[39] Honda K, Taniguchi T (2006) IRFs: master regulators of signalling by Toll-like receptors and cytosolic pattern-recognition receptors. Nature Rev Immunol 6: 644–658. 16932750 10.1038/nri1900

[40] Sentsui H, Takami R, Nishimori T, Murakami K, Yokoyama T, et al (1998) Anti-viral effect of interferon-alpha on bovine viral diarrhea virus. J Vet Med Sci 60: 1329–33. 9879534 10.1292/jvms.60.1329

[41] Panigrahi P, Mohanty SB, Maheshwari RK, Friedman RM (1988) Effect of cloned human interferon-alpha 2a on bovine parainfluenza-3 virus. Brief report. Arch Virol 98: 107–15. 2829793 10.1007/BF01321011

[42] Uematsu S, Akira S (2007) Toll-like receptors and Type I interferons. J Biol Chem 282: 15319–15323. 17395581 10.1074/jbc.R700009200

[43] Buza J, Benjamin P, Zhu J, Wilson HL, Lipford G, et al (2008) CD14+ cells are required for IL-12 response in bovine blood mononuclear cells activated with Toll-like receptor (TLR) 7 and TLR8 ligands. Vet Immunol Immunopathol 126: 273–282. 18789542 10.1016/j.vetimm.2008.08.001

[44] Hart OM, Athie-Morales V, O’Connor GM, Gardiner CM (2005) TLR7/8-mediated activation of human NK cells results in accessory cell-dependent IFN-gamma production. J Immunol 175: 1636–1642. 16034103 10.4049/jimmunol.175.3.1636

[45] Filippi CM, von Herrath MG (2008) IL-10 and the resolution of infections. J Pathol 214: 224–230. 18161757 10.1002/path.2272

[46] Brooks DG, Trifilo MJ, Edelmann KH, Teyton L, McGavern DB, et al (2006) Interleukin-10 determines viral clearance or persistence in vivo. Nature Med 12: 1301–1309. 17041596 10.1038/nm1492 PMC2535582

[47] Fiorentino DF, Zlotnik A, Vieira P, Mosmann TR, Howard M, et al (1991) IL-10 acts on the antigen-presenting cell to inhibit cytokine production by Th1 cells. J Immunol 146: 3444–3451. 1827484

[48] Harrison SM, Tarpey I, Rothwell L, Kaiser P, Hiscox JA (2007) Lithium chloride inhibits the coronavirus infectious bronchitis virus in cell culture. Avian Pathol 36: 109–114. 17479370 10.1080/03079450601156083 PMC7154305

[49] Franchimont D, Martens H, Hagelstein MT, Louis E, Dewe W, et al (1999) Tumor necrosis factor alpha decreases, and interleukin-10 increases, the sensitivity of human monocytes to dexamethasone: potential regulation of the glucocorticoid receptor. J Clin Endocrinol Metab 84: 2834–2839. 10443688 10.1210/jcem.84.8.5931

[50] Jagtap SP, Rajak KK, Garg UK, Sen A, Bhanuprakash V, et al. (2012) Effect of immunosuppression on pathogenesis of peste des petits ruminants (PPR) virus infection in goats. Microb. Pathogenesis. 52, 217–226. 10.1016/j.micpath.2012.01.003 22248720

[51] Goyal S, Dubey PK, Tripathy K, Mahajan R, Pan S, et al (2012) Detection of polymorphism and sequence characterization of Toll-like receptor 7 gene of Indian goat revealing close relationship between ruminant species. Anim Biotechnol 23: 194–203. 22870874 10.1080/10495398.2012.684417

[52] Xu Y, Tao X, Shen B, Horng T, Medzhitov R, et al (2000) Structural basis for signal transduction by the Toll/interleukin-1 receptor domains. Nature 408: 111–115. 11081518 10.1038/35040600

[53] Banerjee P, Gahlawat SK, Joshi J, Sharma U, Tantia MS, et al (2012) Sequencing, Characterization and Phylogenetic analysis of TLR genes of *Bubalus bubalis.* . DHR-IJBLS 3: 137–158.

[54] Dhinakar Raj G, Nachimuthu K, Mahalinga Nainar A (2000) A simplified objective method for quantification of peste des petits ruminants virus or neutralizing antibody. J Virol Methods 89: 89–95. 10996642 10.1016/s0166-0934(00)00206-8

[55] Gopinath VP, Raj GD, Raja A, Kumanan K, Elankumaran S (2011) Rapid detection of Newcastle disease virus replication in embryonated chicken eggs using quantitative real time polymerase chain reaction. J Virol Methods 171: 98–101. 20951166 10.1016/j.jviromet.2010.10.007

