# Correction: Toll-Like Receptor Responses to *Peste des petits ruminants* Virus in Goats and Water Buffalo

**DOI:** 10.1371/journal.pone.0117886

**Published:** 2015-01-29

**Authors:** The PLOS ONE Staff

The last author’s name appears incorrectly. The correct name is Subbiah Elankumaran. Initials referring to this author (“ES”) should appear as “SE”. The competing interests statement should read: The authors confirm that the corresponding author Subbiah Elankumaran is an academic editor for PloS One. This does not alter the authors’ adherence to PLOS ONE Editorial policies and criteria.
